# Utilization of industrial waste materials for the preparation of wollastonite by temperature-induced forming technique

**DOI:** 10.1038/s41598-024-71243-3

**Published:** 2024-09-18

**Authors:** H. H. Abo-Almaged, Rehab E. A. Ngida, N. A. Ajiba, H. E. H. Sadek, R. M. Khattab

**Affiliations:** 1 Refractories, Ceramics, and Building Materials Department, National Research Centre (NRC), Dokki, Giza, 12622 Egypt; 2 Pharos University, Canal El Mahmoudia Street, Smouha, Alexandria, Egypt

**Keywords:** Wollastonite, Bypass, Silica fume, Temperature-induced forming technique, Environmental sciences, Chemistry, Materials science

## Abstract

The study focuses on synthesizing wollastonite using bypass and silica fume waste materials as starting materials. The novelty of this work is the utilization of temperature-induced forming technique for the synthesis of wollastonite. Bypass and silica fume are mixed with various CaO/SiO_2_ ratios and then cast and fired at temperatures ranging from 900 to 1200 °C. Rheological properties and zeta potential are characterized for the slurries to optimize the dispersant percentage. The fired samples' phase composition, structure properties, apparent porosity, linear shrinkage, and compressive strength are characterized. Results show that the sample with a CaO: SiO_2_ ratio of 1:1.45 is the optimum composition for forming mainly pure β-wollastonite at 1100 °C, which changed into pseudo-wollastonite at about 1150 °C. The best physical and mechanical properties are obtained at 1170 °C, including apparent porosity of 8%, bulk density of 2.2 g/cm^3^, linear shrinkage of 13%, and compressive strength of 40 MPa, which widens its ceramic applications.

## Introduction

Waste is described as an unwanted or undesired material, depending on the type of material, and might include materials left over from production processes or family and community activities^1^. Recycling manufacturing wastes is well thought out to be a big concern, both financially and environmentally. The rapid population development and rise in human prosperity have led to increased industrial waste, becoming a significant social and environmental issue. Managing industrial waste has become the most important environmental concern in many developing nations. Human activity mostly produces solid waste, including construction and technological advancement. These wastes result in various intricate issues about storage, transportation, and contamination of the environment or atmosphere. The key to having a clean and safe environment will be using manufacturing waste to create useful resources using green chemical techniques^2–4^.

Wollastonite emerged to overcome waste buildup and create an appropriate precursor for the tile sector. Natural wollastonite has the unique property of minimizing changes in dimensions in ceramic goods' firing and drying processes. Wollastonite finds applications in making heat-insulating ceramics, packaging, lining for foundries, and filling paints and polymers in the metallurgical and automotive industries^5^. Wollastonite is currently in high demand worldwide, especially in areas where asbestos has been replaced, which is detrimental to human health. It is used in the ceramics industry to create hygienic porcelain, earthenware, and special electrotechnical porcelain with minimal dielectric losses. Wollastonite is used novelly in the bioactive ceramic industry, functioning as a bioglass for bone replacement and covering applications^5–7^.

Wollastonite is characterized by a theoretical composition of 52 weight percent SiO_2_ and 48 weight percent CaO, and it is identified as calcium metasilicate with the chemical formula CaSiO_3_^8–10^. Wollastonite is rarely discovered in its pure form and has trace amounts of titanium oxide, manganese oxide, magnesium oxide, iron oxide, and other hazardous contaminants. It is most frequently found as a metamorphic deposit in contact between limestone and garnet, igneous rocks, calcite, diopside, quartz, and epidote^8,11,12^.

Wollastonite exhibits three distinct polymorphic forms: the low-temperature triclinic form [1T], the monoclinic form, commonly referred to as para-wollastonite [2M], and the high-temperature pseudo-wollastonite, which occasionally occurs in its pseudo-hexagonal form in nature. The transformation from low-temperature to high-temperature forms occurs at 1125 °C^5,8,13,14^.

Much work has been done recently to create synthetic wollastonite by solid-state reaction with biowaste as the raw material^15^. For instance, Phutthawong et al.^16^ discovered that the formation of wollastonite increases with grinding time, with the ideal duration being 7 h at a calcination temperature of 1000 °C for 2 h. Wollastonite combines 1:1 molar ratios of silica, rice husk ash, and snail shells (*Pomacea canaliculata*). Rashid et al.^17^ also employed the same procedure, using 99.56 weight percent silica sand and CaO from limestone to create the β-CaSiO_3_ phase at 1050 °C sintering temperature, subsequently converting this phase to α-CaSiO_3_ at 1150 °C sintering temperature. Sintering at 950 °C and 1200 °C produced single-phase β-CaSiO_3_ and α-CaSiO_3_ with an average particle size of 29–50 nm using calcium nitrate and fumed silica as raw materials, synthesized using the solid-state technique^18^. Meanwhile, β-CaSiO_3_ and α-CaSiO_3_ are the wollastonite crystals that develop around 1100 °C and 1300 °C, respectively, according to a study by Nizami^19^. Furthermore, using the solid reaction method, wollastonite crystals with a density of 1.98 g/cm^3^ and a sintering temperature of 1250 °C were produced^20^.

Cement is one of the most astounding binding substances. Over the world, it is manufactured in vast amounts. One industry that generates a substantial amount of solid waste is cement manufacturing. These wastes must be controlled to create a secure and hygienic environment^2–14,21^. Egypt has many cement factories that make a lot of waste. The composition of the source material, gas flow rate, and technique type significantly influence the chemical composition of the bypass byproduct. Bypass frequently contains alkali sulfates, halides, clinker dust, unreacted raw feed, and other volatile components^2,22–25^. About one million tonnes of bypass cement kiln dust (BCKD) are produced annually from the cement industry in Egypt. Recycling these wastes is advised rather than disposing of them in landfills that pollute the land, water, and air. The various wastes used to make roof tiles in place of certain clay are displayed at the following points^26^: A byproduct of the production of elemental silicon, silica fume, is produced in electric arc furnaces by a carbothermic reduction process. When temperatures rise above 1800 °C, silicon monoxide gas escapes and oxidizes in the atmosphere to produce thin, amorphous silica particles. A gas cleaning system gathers condensed silica with dust and other phases^27–31^. Silica fume is an amorphous SiO_2_ with a chemical composition of > 90% silica and a particle size of 0.1 microns^32,33^. Around 18,000 tonnes of silica fume are generated yearly in Egypt as a waste byproduct^34^.

In this work, wollastonite production is performed by using the bypass and silica fume to reduce the final product's cost and eliminate the negative effects of these wastes on the environment. Wollastonite has been prepared using a variety of techniques, such as the solid reaction method^1,17,35^, the sol–gel method^36^, microwave methods^37^, and hydrothermal methods^37–39^. The most common technique is the solid-state reaction method, which yields samples with large grain sizes^40^ and necessitates a long reaction time and high calcination temperature. Despite this, the solid-state reaction method is the most straightforward way to obtain wollastonite material in the ceramic industry.

Using the solid-state method combined with colloidal forming techniques for ceramics offers significant advantages over dry processing techniques due to the potential reduction in defect size. For the near-net-shape forming of intricate ceramic objects, novel forming technologies based on colloidal processing have been created^41–44^. Colloidal forming techniques made it possible to manufacture complex components without machining directly, and it was possible to enjoy homogenous green microstructures and excellent particle packing^45^.

A colloidal technique made ceramic green bodies for slip, tape, and drain casting. Slip casting still needs to be done many more times, and the green body's strength needs to be increased^46^. Numerous near-net shape-forming techniques, such as direct coagulating methods, salt-induced plasticity or short-range steric force, temperature-induced forming, and gel casting, have recently been developed and studied due to the importance of productivity, reliability, economy, and fabricability^46^. To make the process of filling the mold easier, each approach requires a well-dispersed, highly concentrated suspension mold with a manageably low viscosity^45^.

Temperature-induced forming techniques^46^ are among the several approaches for creating wollastonite by solid-state reaction^47^, including freeze casting^48,49^, replication technique^50,51^, and three-dimensional (3D) printing^52^. Temperature-induced formation is less expensive and more accessible than the other techniques; it doesn't require air control, and ess than 0.5% of the organic additives' weight is compared to the ceramic powder's weight^46^.

In temperature-induced forming (TIF), to achieve stability at room temperature, concentrated aqueous suspensions combine a higher molecular weight polymer, specifically polyacrylic acid, with a lower molecular weight dispersion, such as ammonium citrate. Increasing the temperature enhances the dissolution of ceramic particles, facilitating the suspensions to undergo bridging flocculation^53^.

Using a dispersant, the TIF approach stabilizes the ceramic slurry through an electric double layer at room temperature. When added, the polymer forms a bridging network (gel), and the temperature is raised^54–57^. Yang et al. investigated the gelation behavior of the Al_2_O_3_ aqueous suspension during TIF. They discovered that adding polyacrylic acid (PAA) caused the viscosity of the alumina suspension to rise with temperature^58^. In a different investigation, the same authors used the TIF approach with 0.03 weight percent PAA and 0.4 weight percent ammonium citrate tribasic (ACT) to successfully shape complicated alumina pieces with a satisfactory surface polish and green density of 65%^57^. Zirconia green bodies with 0.4 weight percent ACT were prepared by Ewais et al. using TIF. The TIF green bodies had a homogeneous microstructure and good compaction^46^.

Thus, this work concerns preparing wollastonite ceramic materials using bypass and silica fume based on an advanced method called temperature-induced forming. Then, the rheological behaviors, phase composition, microstructure, and physical and mechanical properties are studied to prepare wollastonite with good technological properties. The obtained wollastonite can be used to substitute granite and natural marble in various applications, such as ceramics, architecture, and construction materials like floor materials^59^. The obtained wollastonite can be used in various applications, such as ceramics, architecture, and construction materials like floor materials, as a substitute for granite and natural marble^59^.

## Materials and experimental method

### Starting materials

Alexandria Refractories supplied the starting materials for this work for bypass, while the silica fume was provided by the Egyptian Ferro-alloys company located in Edfu, Aswan, Egypt. The chemicals utilized in this study included ammonium citrate tribasic (dispersant, ACT, Alfa Aesar; USA, 97) and polyacrylic acid (binder, PAA, Alfa Aesar; USA, polyacrylic acid 25% sol. in water). XRF determines the chemical composition of the silica fume and bypass material. The phase compositions, particle sizes, and microstructures are determined by X-ray diffraction (XRD), transmission electron microscope (TEM), and scanning electron microscope (SEM), respectively. A sieving analysis was carried out to determine the silica fume and bypass particle size distribution.

### Experimental method and characterizations

#### Rheological and Zeta potential measurement of ceramic slurries

Before casting the ceramic samples, the ceramic slurries' zeta potential and rheological behavior are assessed to determine the ideal slurry composition. Zeta potential was measured by Zeta Sizer 2000, Malvern (Germany). 5 vol.% solid loading of powders with various percentages of the dispersant (ACT) (0, 0.3, 0.6, 1, and 1.5 wt% based on powder) were used to assess the zeta potential (based on powder) and represented as (w, w0.3 ACT, w0.6 ACT, w1 ACT and w1.5 ACT). For 24 h, the slurry was continuously stirred. This slurry was diluted from 0.4 to 200 mL; as a result, the measurement's solid loading was 0.01 vol.%.

Viscosity measurements were used to determine the rheological characteristics. The viscosity measurement was carried out with MCR301 viscometer with a shear rate varying from 0.05 to 100 s^−1^. Tables 1 and 2 show the composition of samples used to study the rheological behavior, measure shear stress, and measure viscosity at a shear rate of 100 s^−1^. As depicted in Tables 1 and 2, shear stress and viscosity measurements were taken on eight samples. These slurries include metal oxides with weight percentages ranging from 55 to 70%, water contents from 44 to 30 wt%, two dispersants (0.6 and 1 weight percent based on powder), and a small amount of polyacrylic acid (0.06 weight percent) to optimize the dispersant percentage.

**Table 1:** Composition of slurries based on using 0.6 wt% of ACT.

Powder (wt%)	Silica fume (wt%)	Bypass (wt%)	ACT (wt% based on powder)	PAA (wt%)	H_2_O (wt%)
55	16.14	39.63	0.6	0.06	44.17
60	17.38	42.65	0.6	0.06	39.91
65	18.84	46.10	0.6	0.06	35.00
70	20.60	49.34	0.6	0.06	30.00

**Table 2:** Composition of slurries based on using 1 wt% of ACT.

Powder (wt%)	Silica fume (wt%)	Bypass (wt%)	ACT (wt% based on powder)	PAA (wt%)	H_2_O (wt%)
55	16.14	39.63	1	0.06	44.17
60	17.38	42.65	1	0.06	39.91
65	18.84	46.10	1	0.06	35.00
70	20.60	49.34	1	0.06	30.00

### Preparation of samples by temperature-induced forming (TIF) method

**Fig. 1:**
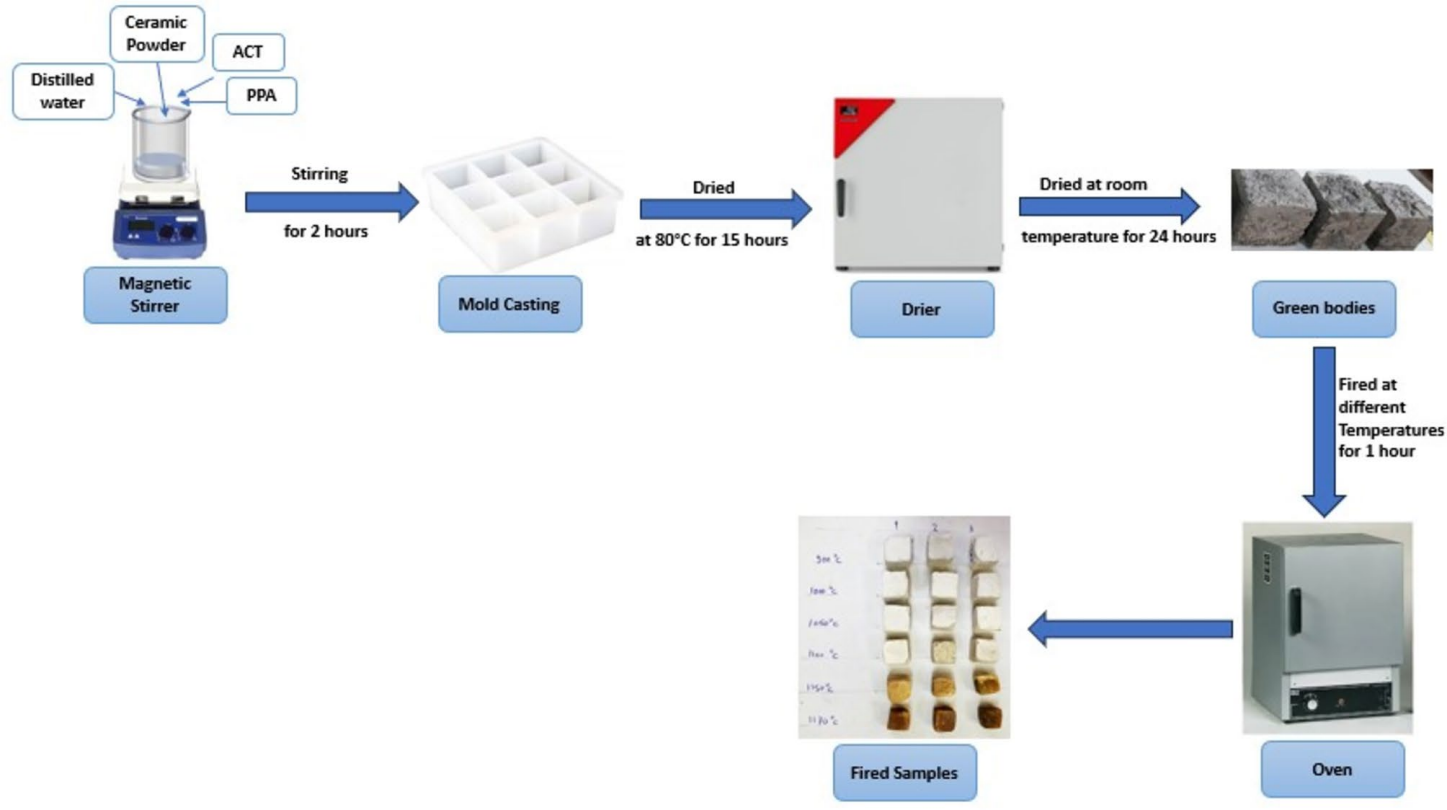
Schematic diagram for samples prepared by the temperature-induced-forming method.

According to the viscosity measurement and zeta potential that are shown later in section "Results and discussion", the optimum weight percentage of selected ceramic oxide is 65 wt% with 0.6 wt% of ACT. The ceramic powder mixes were shaped using the temperature-induced forming (TIF) process (Fig. 1). Usually, to create an aqueous ceramic slurry, 65 weight percent of the ceramic powder mixture (18.85 wt% of silica fume and 46.10 wt% of bypass) was added to 35 wt% water, along with ammonium citrate tribasic (ACT = 0.6 wt%, depending on powder), and poly acrylic acid (PAA = 0.06 wt%). The slurry was then mechanically mixed with a Daihan Scientific mixer at 200 rpm at room temperature for 2 h. During the procedure, the gelling agents and dispersant were absorbed onto the surface of the ceramic slurry, resulting in the formation of an electrical double-layer (EDL) that had a steric impact^60^. After casting the composite slurries in an acrylic mold with 1.5 cm × 1.5 cm × 1.5 cm, the temperature was raised to 80 ºC for 15 h to induce gel formation and consolidate the green bodies. To reduce the inhomogeneous shrinkage, plastic sheets were used to cover the molds, slowing down the evaporation of water. After the forming process, the samples were dried at room temperature for 24 h. The samples were then fired for 1 h at 900, 1000, 1050, 1100, 1150, 1170, and 1200 ºC to track the phase compositions. The heating rates are divided into three stages. The temperature is raised from 0 to 300 °C in 20 min to prevent hydration of CaO, followed by an increase in the temperature from 300 to 700 °C over 3 h to burn out the organic matter slowly. Finally, the temperature is raised to the final firing temperature (900, 1000……1200 °C) in 1 h. Then, the furnace was switched off, and the samples were permitted to cool inside it to room temperature.

The other three slurry compositions containing excess silica, as seen in Table 3, are prepared. These slurries are designed according to the same procedure as the above slurry by TIF by changing the weight percentage of the CaO and SiO_2_ ratios to optimize the wollastonite formation. The three mixes were fired at various temperatures ranging from 900 ºC to 1170 ºC.

**Table 3:** Composition of prepared mixes with various CaO/SiO_2_ ratios.

Powder (wt%)	CaO: SiO_2_ (wt ratio)	Silica fume (wt%)	Bypass (wt%)	PAA (wt%)	H_2_O (wt%)
65	1: 1	18.84	46.10	0.06	35.00
65	1: 1.17	20. 42	44.52	0.06	35.00
65	1: 1.25	21.62	43.32	0.06	35.00
65	1: 1.45	24.41	40.53	0.06	35.00

### Characterization methods of the starting materials and fired samples

The chemical composition of the representative starting materials was quantitatively assessed using wavelength-dispersive X-ray fluorescence (AXIOS, WD-XRF Sequential Spectrometer from Panalytical, 2005). The X-ray powder diffraction (XRD) technique was employed to examine the phase composition of both the starting materials and fired samples. The analysis was conducted at room temperature using a diffractometer (Bruker D8 Advance, Germany) equipped with a Ni filter and Cu Kα radiation, with a scan speed of 0.5 min^−1^. The microstructure of both the starting materials and fired samples was analyzed using a Philips XL 30 scanning electron microscope (SEM) instrument from the UK. Transmission electron microscope (TEM) JEM-2100 was used to examine the particle size of the starting materials. The thermogravimetric analysis was performed using BEL-Sorb Max equipment made in Japan.

The apparent porosity and bulk density were assessed through the Archimedes water displacement method, following the guidelines outlined in the American Society for Testing and Materials (ASTM C373-88)^61^. Water is added to three test samples, which are then boiled for two hours. After submerging in water (WI), the saturated samples were weighed again in the air (WS). After being left overnight at 110 °C, the samples were dry-weighted (Wd). The test samples' bulk density (BD) and apparent porosity (AP) were determined using the following formulas (1 and 2):

1 $$Ap = \frac{{\left( {WS - Wd} \right)}}{{\left( {WS - WI} \right)}} \times 100$$

2 $$BD=\frac{Wd}{(WS-WI)} \times \upgamma $$

Where $\upgamma$ is density.

The compressive strength (CCS) of the three fired samples was measured using a hydraulic testing machine (Seidner model, Riedlinger, Germany) with a maximum load capacity of 600 KN (ASTM C133-97)^62^. The compressive strength (CCS) of the test samples was calculated using the formula below (3):

3 $${\text{CCS}} = \frac{{{\text{Stress}}}}{{{\text{Area}}}}$$

## Results and discussion

### Characterization of the starting materials

Table 4 illustrates the chemical composition of silica fume and bypass determined using the XRF method. According to Table 4, silica accounts for more than 92% of the main oxide compositions in silica fume. The predominant phase in the bypass is made primarily of silica and CaO.

**Table 4:** Chemical composition of the starting materials.

Main constituents	Silica fume (wt%)	Bypass (wt%)
SiO_2_	92.46	13.18
TiO_2_	–	0.40
Al_2_O_3_	0.74	8.08
Fe_2_O_3_	2.02	4.41
MnO	0.12	–
MgO	0.42	2.12
CaO	0.33	50.32
Na_2_O	0.37	1.43
K_2_O	0.84	3.33
P_2_O_5_	0.13	0.09
SO_3_	0.35	6.60
Cl	0.08	7.54
LOI	1.88	2.16

**Fig. 2:**
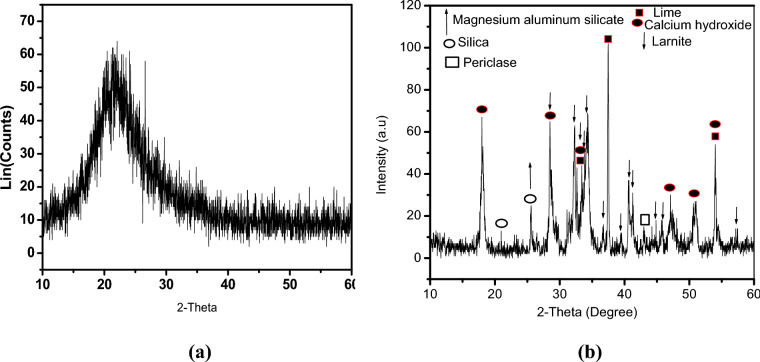
XRD of the starting materials: (**a**) silica fume, (**b**) bypass.

The XRD was utilized to analyze the phase compositions of these materials. The amorphous phase of silica is easily visible for silica fume particles since there are no crystalline peaks other than a faint and extremely broad hump about 21.8, as can be shown in Fig. 2a ^63^. In the case of bypass, the lime (CaO) and hydrated lime (Ca(OH)_2_) were the most prevalent components percentage in bypass dust, followed by Larnite Ca_2_SiO_4,_ as shown in Fig. 2b. Other small components such as calcite, quartz, periclase, MgO, and Al_2_O_3_ were also present. The secondary phases, quartz, and calcite, are derived from untreated raw materials.

**Fig. 3:**
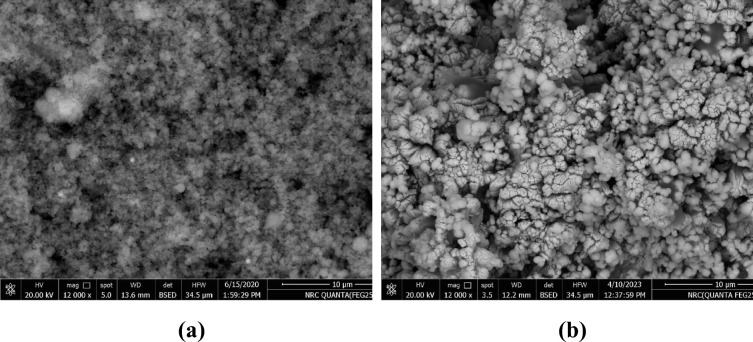
SEM of the starting materials: (**a**) silica fume, (**b**) bypass.

Figure 3 displays the SEM of silica fume and bypass. The spherical particles of silica fume (Fig. 3a) range in average diameter from 0.2 µm to around 2 µm. The SEM of bypass (Fig. 3b) is a heterogeneous combination of particles with different morphologies, including large and tiny particles. Small dust particles are typically grouped in clusters of 1–3 µm (Fig. 3b). The charges cause some small particles to adhere to the surfaces of the giant particles. The size of the little particles joined to the giant particles varies. Still, they are often sub-micron-sized (Fig. 3b). The little brightness that can be seen in the SEM micrographs of the microscopic particles is a result of electron charging during imaging. The fact that these particles are charged suggests that they have an electrical charge that enables them to adhere to the giant particles^64^.

**Fig. 4:**
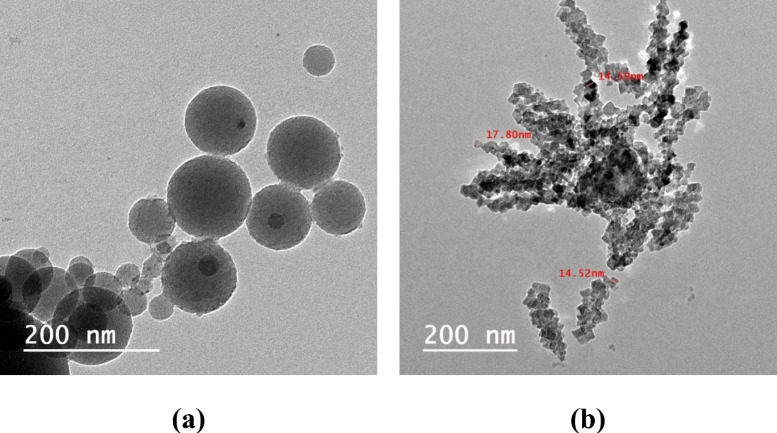
TEM of the starting materials: (**a**) silica fume, (**b**) bypass.

Figure 4 displays the raw materials' TEM characterization. According to the TEM investigation (Fig. 4a), silica fume still has its original spherical shape. According to TEM results for bypass (Fig. 4b), the diameters of the particles in those samples, which were shaped like plates, ranged from 10 to 95 nm.

Sieving analysis was conducted to determine the particle size distribution for silica fume and bypass, as shown in Table 5. It was found that the bypass is finer than silica fume. As seen in Table 5, the highest percentage of particles at the 200 micron size is for silica fume, while the highest percentage of particles in the 75–100 micron size range is for bypass.

**Table 5:** Sieving analysis results for silica fume and bypass.

Size (µ)	Silica fume (wt%)	Bypass (wt%)
+ 200	37.80	0.80
− 200 + 150	25.57	5.60
− 150 + 125	8.55	12.60
− 125 + 100	2.35	27.68
− 100 + 75	17.00	40.80
− 75 + 63	7.13	10.36
− 63 + 40	1.44	2.12
− 40	0.16	0.04
Total	100 (%)	100 (%)

### Zeta potential

**Fig. 5:**
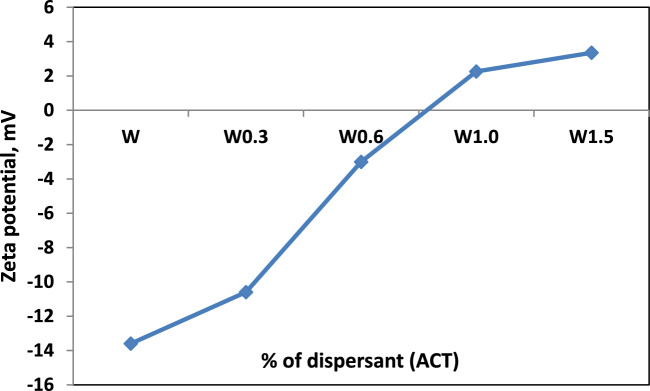
Zeta potential characterization.

The zeta potential measurements for the suspended liquids with various amounts of ACT are shown in Fig. 5. It is observed that the suspension of the powder without dispersant shows a low zeta potential value. A low zeta potential value shows that the powder does not disperse well without a dispersant. The zeta potential significantly increases with the addition of ACT. The iso-electric point (IEP) shifts to the positive region, resulting in a higher zeta potential (3.35 mV). It is indicated that the addition is necessary for stabilizing the powder.

The stabilization mechanism can be illustrated as follows: the dissociation of dispersant agents in water containing carboxyl and ammonium groups occurred. This produces a positive charge (ammonium group) and a negative charge (carboxyl group). The negative charge of the carboxyl group is absorbed on the positively charged calcium oxide surface. In contrast, the positive charge of the ammonium group is adsorbed on the negatively charged silica surface, leading to good stabilization of the slurry.

### Viscosity

**Fig. 6:**
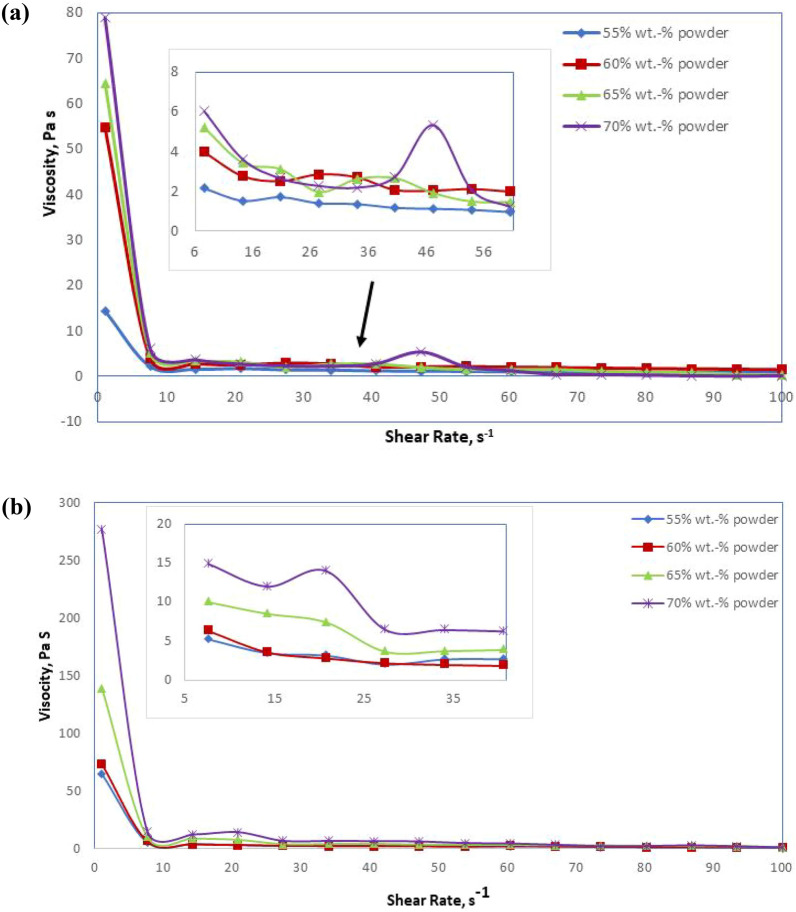
Rheological characteristics based on using: (**a**) 0.6.wt% of dispersant, (**b**) 1wt% of dispersant.

According to the results of the zeta potential, two percentages of dispersant 0.6 and 1 wt% based on powders are selected to show the effect of each percentage value of dispersant on the ceramic slurry, as seen in Fig. 6. It is noted that the viscosity rises with the increase in weight percentage of ceramic oxides from 55 to 70 wt%. The suspensions approach a constant viscosity at high shear rates and exhibit shear thinning behavior. When the amount of ceramic oxides increases up to 65 weight percent, both the degree of shear thinning and the viscosity at high shear rates rise. Because thermal motion outweighs viscous forces at low shear rates, the suspension structure approaches the equilibrium structure at rest. Shear thinning happens at greater shear rates because the viscous forces act more strongly on the suspension structure^65^. It was found that certain regions showed shear thickening when the ceramic oxide level was raised to 70%. watery.

At low shear rates, colloidally stable suspensions exhibit a rather robust shear thinning behavior. This is followed by discontinuous shear thickening at ceramic oxide concentrations of 70%. The shear thickening can be explained as the result of a particle structural order–disorder transition. At the critical shear rate, the ordered layers that emerge in the shear thinning zone disintegrate into a less ordered structure. Because of particle “jamming,” this less ordered structure loses more energy during flow, increasing viscosity. Most scientists believe that shear thickening only happens in concentrated suspensions of solid particles that don't aggregate^65–67^. It has been discovered that variables such as the medium's viscosity, tile particle size, and the strength of the interparticle repulsion regulate when shear thickening begins^65–67^. Shear thickening may be linked to the creation of clusters due to hydrodynamic forces, according to a recent study that integrated rheology with suspension structure analysis^65^.

According to Xu et al., the viscosity required for colloidal forming must be less than 1 Pa s at a shear rate of 100 s^−1^. This viscosity is low enough for casting and degassing^67^. Therefore, a powder percentage of 65 wt% is chosen in subsequent experiments. In comparison with the slurry containing 1 wt% of dispersant, it is observed that these slurries are more viscous, especially at 0 s^−1^ shear rate. This means an excess amount of dispersant that does not adsorb onto the particle's surface. Thus, 0.6 wt% of dispersant is suitable for slurry preparation.

### DTG and TG analysis

**Fig. 7:**
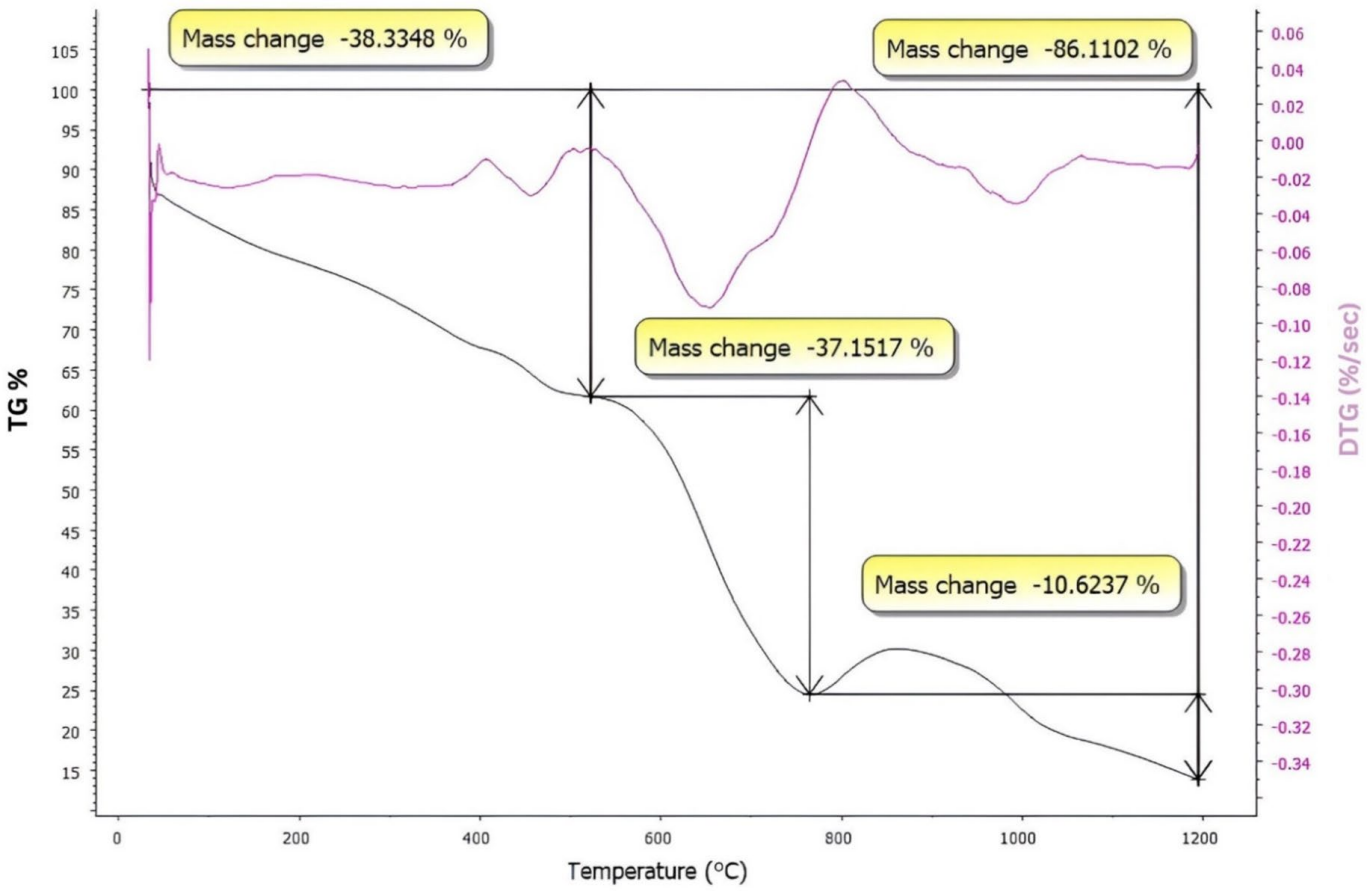
Thermal analysis (DTG) and thermogravimetric analysis (TG) for the green sample.

Figure 7 displays the green sample's profiles for differential thermal analysis (DTG) and thermogravimetric analysis (TG). It was noted that there are distinct stages of thermal breakdown. The DTG analysis reveals two distinct peaks, one between 35 and 145 °C and the other between 400 and 500 °C. Additionally, there is a 38% decrease in weight, explained by the vaporization of certain organic debris, interior water, and absorbed water. After that, a sharp increase between 510 and 780 °C in the DTG analysis shows a continual weight loss of 37% upon heating to 780 °C. The delayed elimination of chemically absorbed water, PAA, and TAC is the reason for this^34^. In DTG, a peak appears between 930 and 1030 °C, indicating slow weight loss, evident after 800 °C and reaching 10%. The reason for this is that β-wollastonite is forming. Owing to the transformation of β-wollastonite into α-wollastonite, the steady state is reached after 1030 °C^15,34^.

### XRD of fired samples

**Fig. 8:**
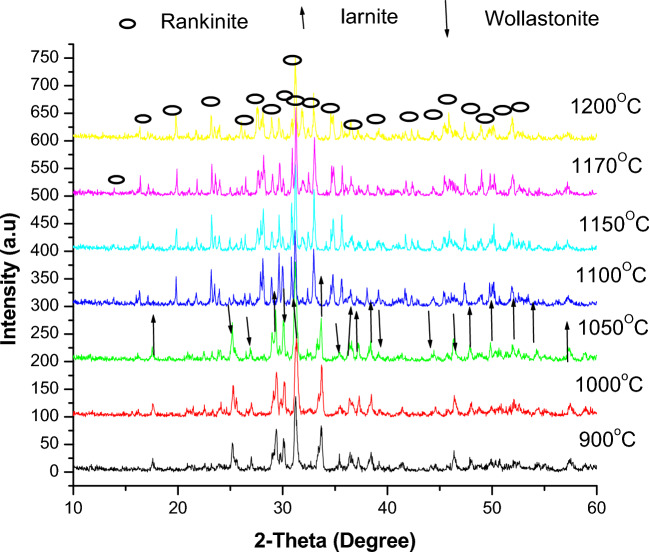
XRD of fired samples with CaO/SiO_2_ ratio = 1:1.

The XRD results in Fig. 8, depicting the work to synthesize wollastonite from bypass waste and silica fumes, show that the stoichiometric composition of lime and silica (CaO/ SiO_2_ ratio = 1:1) gave a mixture of larnite (PDF card No.#77-0388) and wollastonite (PDF card No. 76-1846) for the samples fired at 900, 1000 and 1050 ºC. Moreover, above 1050 ºC, the larnite started to disappear, and the wollastonite phase intensity peaks decreased. Both phases changed into rankinite (PDF card No. 76-0623) for the samples fired at 1150, 1170, and 1200 ºC, as seen in Fig. 8.

The mechanism of the reaction can be explained as follows (Eqs. 4–11)^68^: when a sample is fired between 900 and 1000 °C

4 $$ {\text{3CaO }} + {\text{ 2SiO}}_{{2}} \to {\text{ 2CaSiO}}_{{3}} \left( {{\text{wollastonite}}} \right) \, + {\text{ CaO}} $$

5 $$ {\text{3CaO }} + {\text{ 2SiO}}_{{2}} \to {\text{ CaSiO}}_{{3}} + {\text{ Ca}}_{{2}} {\text{SiO}}_{{4}} \left( {{\text{larnite}}} \right) $$

6 $$ {\text{CaSiO}}_{{3}} + {\text{ CaO }} \to {\text{ Ca}}_{{2}} {\text{SiO}}_{{4}} $$

The proportions of unreacted CaO and silica reduced with the further development of larnite (C2S) and wollastonite (CS) when the calcination temperature was raised to 1050 °C. At the same time, the formation of akermanite and bredigite Ca_7_Mg(SiO_4_)_4_ may have begun earlier, at 900 °C.

7 $$ {\text{2CaSiO}}_{{3}} + {\text{ MgO }} \to {\text{ Ca}}_{{2}} {\text{MgSi}}_{{2}} {\text{O}} $$

8 $$ {\text{MgSiO}}_{{3}} + {\text{ 2SiO}}_{{2}} + {\text{ Ca}}_{{2}} {\text{SiO}}_{{4}} + {\text{ 5CaO }} \to {\text{ Ca}}_{{7}} {\text{MgSi}}_{{4}} {\text{O}}_{{{16}}} $$

**Fig. 9:**
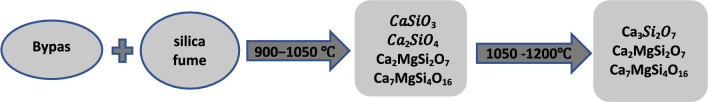
Summary of chemical reactions occurring during heat treatment.

Akermanite formation is thought to occur in the MgO-CaSiO_3_ (pseudo) binary system^69^. On the other hand, transitional intermediate compounds like wollastonite, larnite, merwinite, and calcium magnesium silicate^70^ are formed during the bredigite formation process. Considering their small presence in the finished product, these Ca and Mg silicates shouldn't impact the final material's characteristics, even though they were created due to the initial mixture's relatively high magnesium content. With increasing the firing above 1050 °C, rankinite (Ca_3_Si_2_O_7_) formation is starting to develop. A further increase of the calcination temperature up to 1200 °C led to full recrystallization of CS and C2S to rankinite (C3S2) (Fig. 9).

9 $$ {\text{3CaSiO}}_{{3}} \to {\text{ Ca}}_{{3}} {\text{Si}}_{{2}} {\text{O}}_{{7}} + {\text{ SiO}}_{{2}} $$

10 $$ {\text{3Ca}}_{{2}} {\text{SiO}}_{{4}} + {\text{ SiO}}_{{2}} \to {\text{ 2Ca}}_{{3}} {\text{Si}}_{{2}} {\text{O}}_{{7}} $$

11 $$ {\text{CaSiO}}_{{3}} + {\text{ Ca}}_{{2}} {\text{SiO}}_{{4}} \to {\text{ Ca}}_{{3}} {\text{Si}}_{{2}} {\text{O}}_{{7}} $$

Wollastonite can only form in pure systems under hydrothermal conditions or when mineralizers are present. Despite the attempt in the current study using bypass and silica fume, pure wollastonite could not be obtained, even when subjected to high firing temperatures of 1200 ºC, as in Ref.^5^. This is consistent with the findings of Balkevich et al.^71^, who found that siliceous limestone at 850 ºC can form wollastonite. They attributed its formation to the fine particle size of the components and intimate mixing. Furthermore, Kotsis and Balogh^72^ documented the formation of wollastonite from silica gel and limestone at 1300 ºC. However, they did not clarify whether the presence of impurities in the limestone was the cause of its existence.

According to Nour et al.^5^ the existence of impurities such as magnesia, alkali (Na_2_O and K_2_O), aluminum oxides, and iron in both wastes promoted the early formation of wollastonite at 900 °C and a liquid phase at approximately 1100 ºC. According to the phase diagram of CaO-SiO_2_, wollastonite formation is expected to occur at around 1450 ºC^73^. However, at around 1100 °C, the liquid phase is formed early because of feldspars' creation and iron oxide's presence during the firing process^74,75^. Since feldspars supply alkaline oxides like Li_2_O, Na_2_O, and K_2_O that help create a low-viscosity liquid at high firing temperatures, they are regarded as fluxing agents^75^.

In general, the stoichiometric composition of the materials contains impurities that exceed approximately 4%, which is even higher than the recommended amount of 3.6% by various authors for the formation of wollastonite^72^. These impurities and the required amount of silica contributed to the formation of a melt, thereby reducing the stoichiometric ratio of 1:1 necessary for forming pure wollastonite as the sole phase. As a result, the crystallization of rankinite occurred alongside very small amounts of pseudo-wollastonite in samples fired at temperatures of 1150, 1170, and 1200 ºC, Fig. 8. To address this issue, it was necessary to increase the proportion of silica to provide the required amount for wollastonite formation. According to Nour et al*.*^5^, the CaO/SiO_2_ ratio of 1:1.125 was identified as the most suitable, as the two higher ratios, 1:1.166 and 1:1.25, led to the presence of cristobalite. In line with Balkevich et al*.*^71^, the stoichiometric composition decreased alongside the formation of (belite) β-2CaO-SiO_2_. However, in the current study, larnite Ca_2_SiO_4_ was formed. An excess of silica is essential to shift the reaction towards wollastonite formation (CaSiO_3_). In this situation, silica reacts directly with the larnite, lime, or rankinite formed, transforming it into wollastonite.

**Fig. 10:**
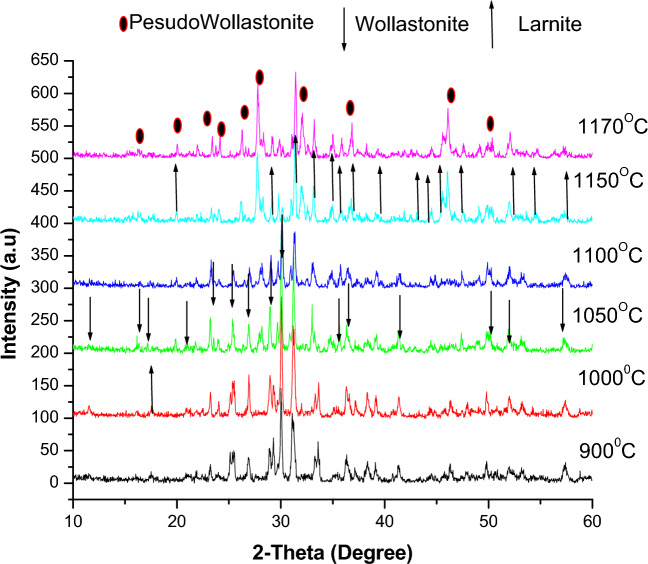
XRD of fired samples with CaO/SiO_2_ ratio = 1:1.17.

**Fig. 11:**
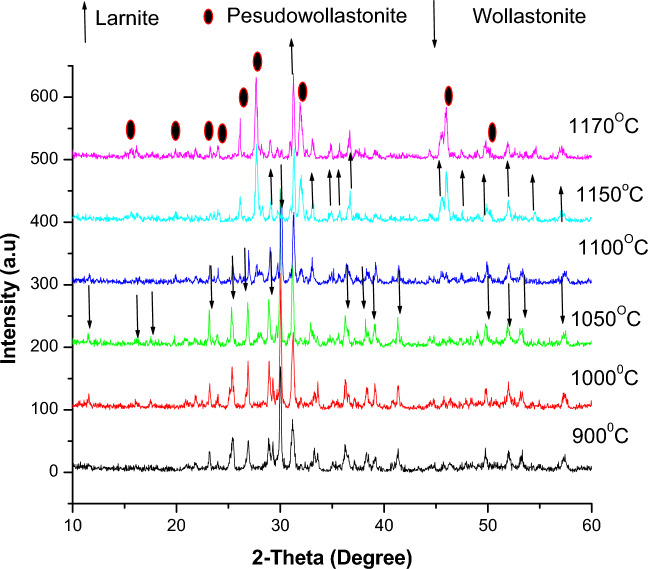
XRD of fired samples with CaO/SiO_2_ ratio = 1:1.25.

**Fig. 12:**
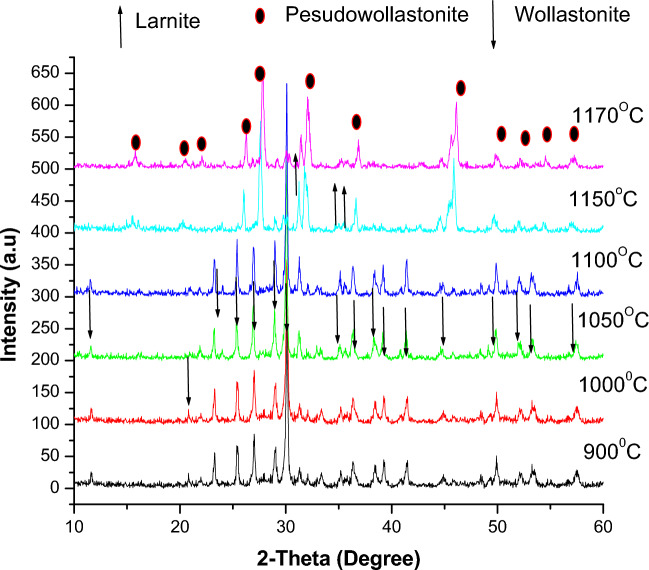
XRD of fired samples with CaO/SiO_2_ ratio = 1:1.45.

Thus, from the above, three other slurries with different CaO: SiO_2_ ratios are prepared, i.e., 1:1.17, 1:1.25, and 1.45, and then the obtained green samples are fired at temperatures ranging from 900 to 1170 ºC. The XRD patterns are shown in Figs. 10, 11, and 12. For samples containing CaO: SiO_2_ ratio of about 1: 1.17, the mixture of larnite and wollastonite was formed after firing at 900, 1000, 1050, and 1100 ºC as seen in Fig. 10. The larnite amount increased with the rise in firing temperature at the expense of wollastonite. Upon raising the temperature above 1100, the wollastonite changes into pseudo-wollastonite, and larnite amounts are increasing continuously until reaching 1170 °C.

With increasing the CaO: SiO_2_ ratio to 1:1.25, as seen in Fig. 11, the larnite content is still high with wollastonite and pseudo-wollastonite at different firing temperatures. However, the wollastonite intensity peaks are higher than the samples of CaO: SiO_2_ ratio of 1:1.17 (Fig. 10), as seen for samples fired at 1100°C.

The best result for well wollastonite formation is indicated after using a CaO: SiO_2_ ratio of about 1:1.45, as seen in Fig. 12. The highest amount of wollastonite is indicated after firing at 1100°C with the least amount of larnite. When the firing temperature is increased above 1100 °C, the wollastonite is converted to pseudo-wollastonite. The pseudo-wollastonite formation increases with increasing firing temperatures to 1170°C. At this firing temperature, pseudo-wollastonite is the main phase, besides the least amount of larnite.

The highly active excess amorphous nano-SiO_2_ and the active CaO derived from the silica fume and bypass exhibit a higher potential for reacting with each other through diffusion reactions. This results in the formation of a pure wollastonite phase in these samples. The pseudo-wollastonite, i.e., α-wollastonite phase, is mostly achieved in samples fired at 1170 °C. When the temperature rises above 1100 °C, a polymorphic transformation reaction occurs in the wollastonite phase, transforming from a low-temperature phase called β-wollastonite to a high-temperature phase called α-wollastonite. The eutectic invariant point in the CaO-SiO_2_ binary system typically occurs around 1500 °C for a 1:1 molar ratio of CaO and SiO_2_. On the other hand, in this study, silica fume and bypass melted at a temperature lower than the eutectic point. This is attributed to both containing more active CaO and SiO_2_ (in amorphous form) than conventional sources. Moreover, the constituents' fineness significantly affects the material's melting temperature. So, the use of micro calcium oxide and nano-silica fume may accelerate the reaction, causing it to melt at a lower temperature than the eutectic invariant point^5,76–78^.

Compare this study to Sk S. Hossain et al.^79^, who create synthetic wollastonite using a cost-effective solid-state method using rice husk ash and chicken eggshells. The peaks of unreacted cristobalite (SiO_2_), minor quantities of larnite (Ca_2_SiO_4_), and β-wollastonite are seen at 1000 °C. After being calcined at 1100 °C, single-phase β-wollastonite is obtained, and larnite and cristobalite are removed through mutual reaction. Furthermore, at high temperatures, the β-wollastonite changes into its polymorphic form, or pseudo-wollastonite, which is the α-wollastonite. The calcined powder consists primarily of the pseudo wollastonite phase at 1200 °C, with the collaborative α and β-wollastonite phases present at 1150 °C.

### SEM of the fired samples

**Fig. 13:**
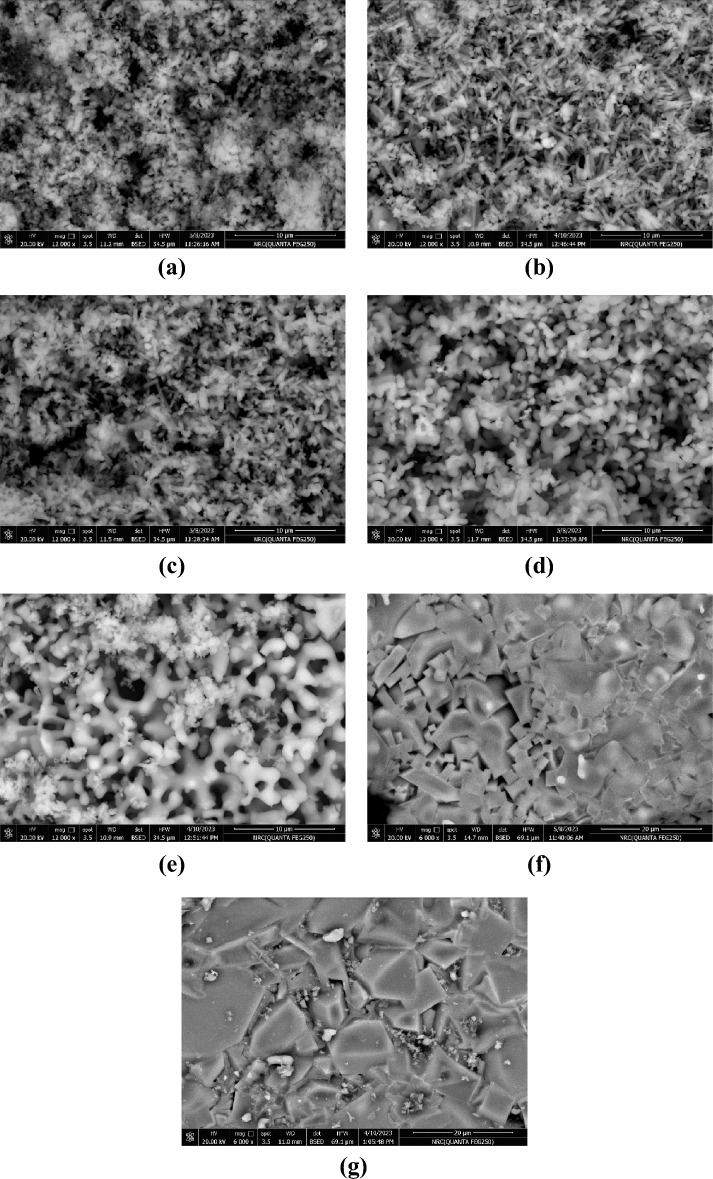
SEM images for samples fired at different temperatures (**a**) 900°C, (**b**)1000°C, (**c**) 1050°C, (**d**) 1100°C, (**e**) 1150°C, (**f**) 1170°C, and (**g**) 1200°C.

SEM for samples fired at various temperatures are seen in Fig. 13. It was found that the samples with CaO: SiO_2_ ratio 1:1 exhibit various morphologies after firing at different temperatures. As seen in Fig. 13a, wollastonite appeared in needle shape at 900 ºC, like in morphology as Reference^80^, and larnite occurred as subspherical grains. As the firing temperature increases to 1050 ºC, the wollastonite content increases and more needle shapes appear with larnite, as seen in Fig. 13b and c. The needle shapes of wollastonite started to disappear at 1100 and 1150 ºC. Moreover, anhedral particle shapes have appeared that are related to the starting of crystallization of rankinite, as seen in Fig. 13d and e. The good crystallization of rankinite as mainly sole phase appeared at 1170 and 1200 °C (Fig. 13f and g), which appeared as gray polygonal shapes like Ref.^68^ in between some rankinite phases appeared in the form of cubic phase. It was observed that the pinholes decreased with the increase in firing temperatures.

**Fig. 14:**
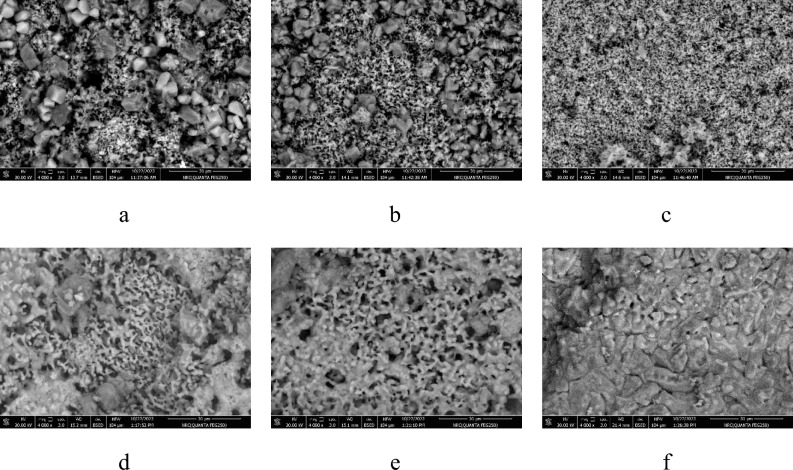
SEM of samples fired at 1050 °C: (**a**) CaO/SiO_2_ = 1:1.17, (**b**) CaO/SiO_2_ = 1:1.25, and (**c**) CaO/SiO_2_ = 1:1.45—samples fired at 1150°C: (**d**) CaO/SiO_2_ = 1:1.17, (**e**) CaO/SiO_2_ = 1:1.25, and (**f**) CaO/SiO_2_ = 1:1.45.

SEM images of some selected samples with varying ratios of CaO/SiO_2_ and fired at 1050 and 1150°C are seen in Fig. 14. It was observed that the samples with CaO/SiO_2_ = 1:1.17 and CaO/SiO_2_ = 1:1.25 are still having larnite (gray cubic shapes) embedded between the needle grains of wollastonite after firing at 1050°C as seen in Fig. 14a and b. Upon raising the CaO/SiO_2_ ratio to 1:1.45 (Fig. 14c), larnite mostly disappeared, and the needle wollastonite grains were the main constituent of the samples. For the samples fired at 1150°C, it was observed that wollastonite disappeared and was replaced by pseudo-wollastonite. Irregular elongated grains are determined for pseudo-wollastonite. Larnite is enlarged and accumulated together, as seen in Fig. 14 (d and e). The larnite is still embedded between the pseudo-wollastonite for CaO/SiO_2_ ratios 1:1.17 and 1:1.25. The pseudo-wollastonite appeared as the main phase with the disappearance of the wollastonite in Fig. 14f. The high compaction and less porosity are observed for samples fired at 1150 °C.

### Physical properties

**Fig. 15:**
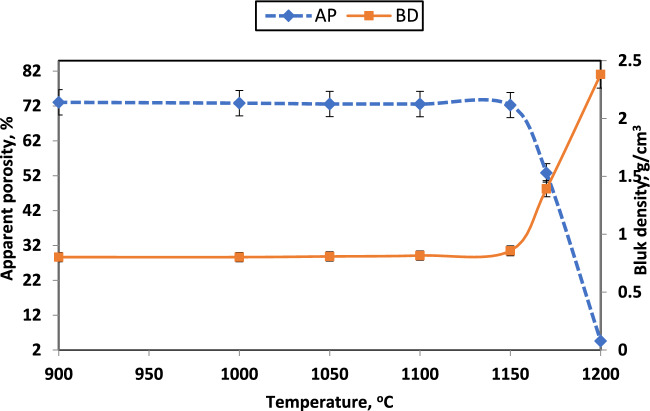
Apparent porosity and bulk density of sample with CaO/SiO_2_ = 1:1 fired at various temperatures.

**Fig. 16:**
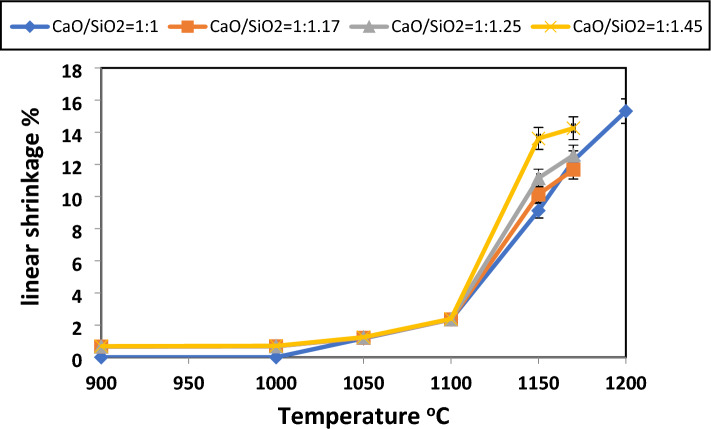
Linear shrinkage of samples with various CaO/SiO_2_ ratios fired at different temperatures.

Physical properties of apparent porosity (AP) and bulk density (BD) are shown in Fig. 15 for samples with CaO/SiO_2_ = 1:1 and fired at various temperatures. It was observed that apparent porosity decreases and bulk density increases with increasing the firing temperature. This finding can be primarily attributed to the similar linear shrinkage observed with the increase in firing temperatures, as seen in Fig. 16. Linear shrinkage was raised from 0% to around 15% when the firing temperature increased from 900 to 1200 °C. The pores in ceramic bodies get filled through ceramic processing as the grains grow throughout the body. The low temperature utilized in this case was insufficient to achieve densification, and consequently, it did not lead to significant closure of the porosity^39^^81^. Thus, the rise in firing temperature has also led to pore closure and improved particle interaction, which increases the shrinkage percentage^82^. In addition, using fine particles expedites the densification process, leading to a reduction in open porosity. This is due to the formation of liquid phases such as calcium aluminosilicates that contain a smaller quantity of liquid phase during firing, consequently decreasing porosity^83^.

Up to 1000 °C, no appreciable shrinkage or expansion behavior is seen for CaO: SiO_2_ = 1:1. However, the graph indicates that the densification of the compacted powders starts at about 1050 °C and that the rate of shrinking rises as the temperature rises. Wollastonite exhibits sintering behavior similar to what was seen in earlier research^79,84,85^. The linear shrinkage values of wollastonite are 0.82, 3.9, and 6.27% for 1100, 1200, and 1250 °C were obtained by^79^. The high powder verification may cause these increases in shrinkage values compared with Hossain et al.^79^.

**Fig. 17:**
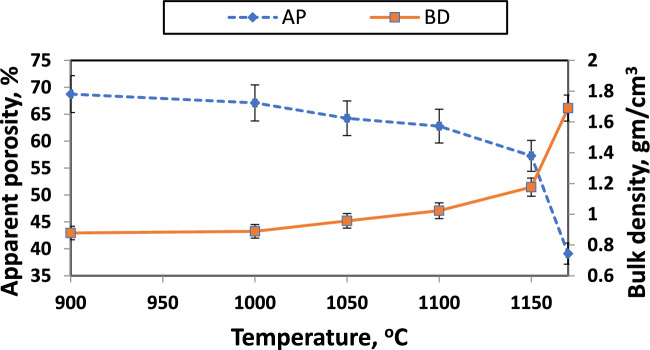
Apparent porosity and bulk density of samples with CaO/SiO_2_ = 1:1.17, fired at various temperatures.

**Fig. 18:**
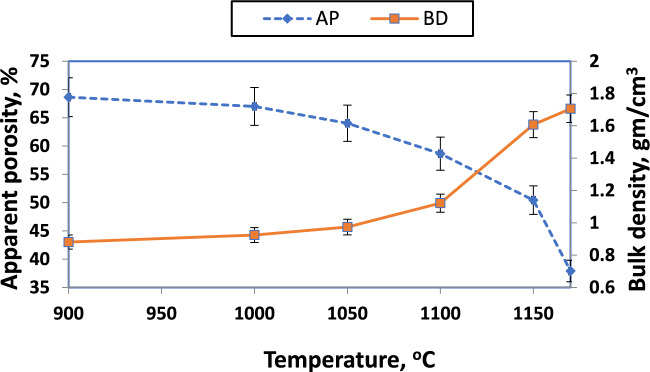
Apparent porosity and bulk density of samples with CaO/SiO_2_ = 1:1.25, fired at various temperatures.

**Fig. 19:**
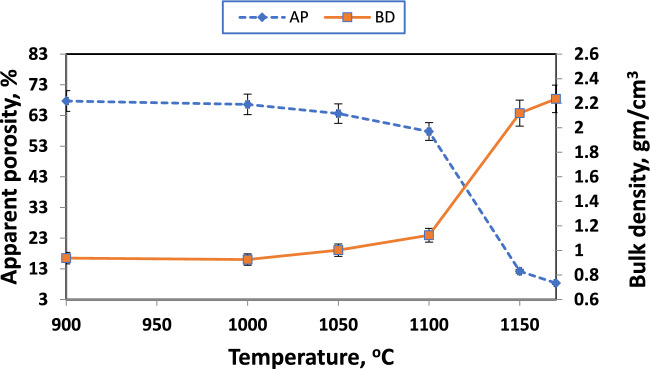
Apparent porosity and bulk density of samples with CaO/SiO_2_ = 1:1.45, fired at various temperatures.

Various calcium-containing materials are one approach for controlling firing shrinkage^57^. Figures 17, 18, and 19 illustrate samples' bulk density and apparent porosity with various CaO/SiO_2_ ratios fired at various temperatures. The linear shrinkage of these samples was observed in Fig. 16 after firing at various temperatures. It was observed that there is an increase in shrinkage and density and a decrease in apparent porosity with increasing the CaO/ SiO_2_ ratios up to 1:1.45.

These results are due to the formation of more wollastonite observed by increasing the silica content in the samples. By developing a greater amount of liquid phase, Wollastonite leads to the closing of pores in samples of CaO/SiO_2_ ratio 1:1.45 than the other samples^86^. This sample exhibits mainly the formation of pseudo-wollastonite (α-wollastonite) with porosity of about 8%, density of about 2.2 g/cm^3^, and linear shrinkage of about 13% at 1170°C. The increased firing temperature has also enhanced linear shrinkage, attributed to pore closure and improved particle interaction^46^.

**Fig. 20:**
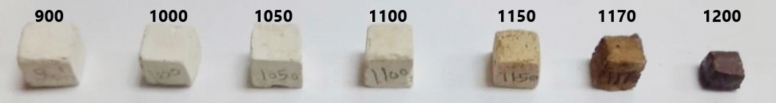
Photo image of samples with a CaO: SiO_2_ ratio of 1:1, fired at different temperatures.

**Fig. 21:**
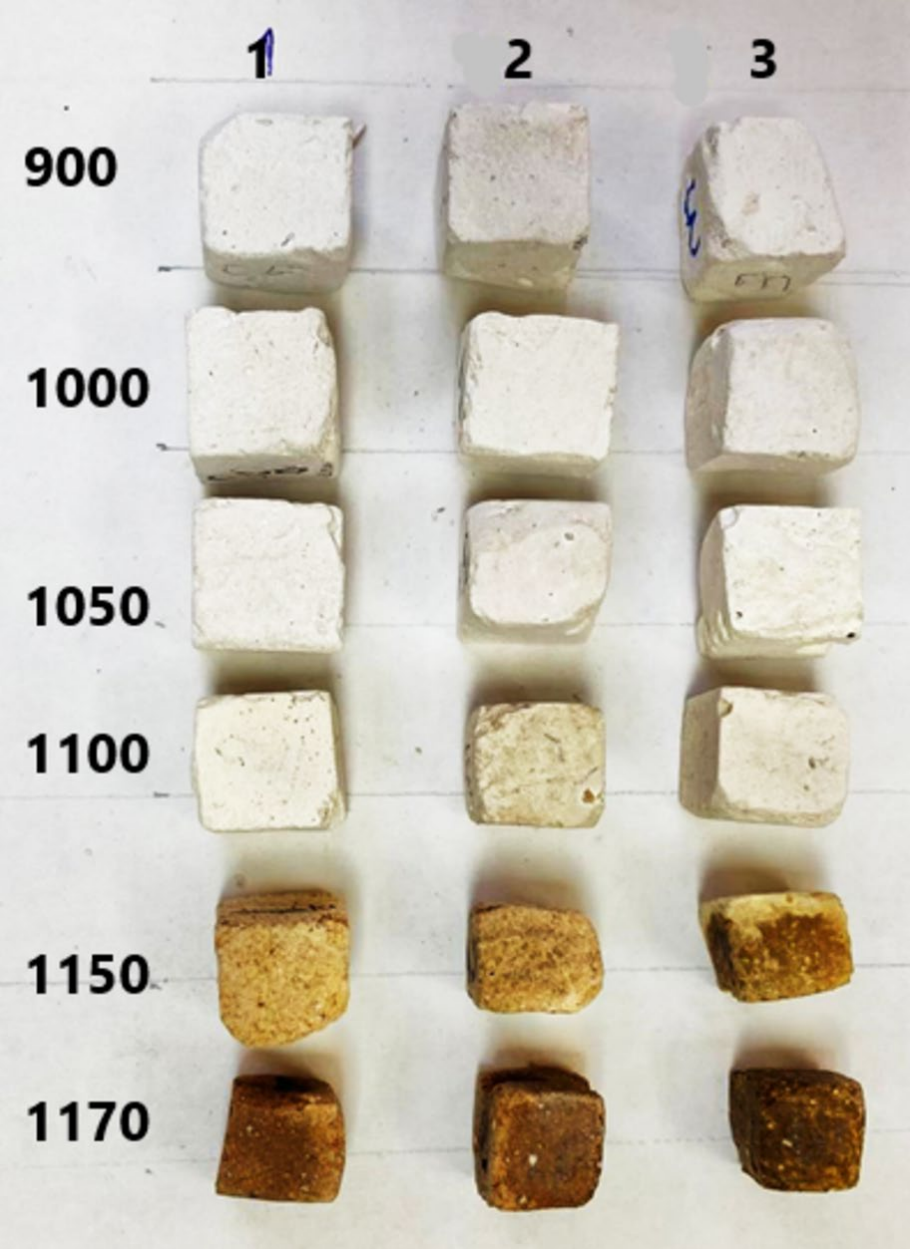
Photo image of samples with different CaO: SiO_2_ ratios (**1** = 1: 1.17,** 2** = 1: 1.25, and **3** = 1: 1.45) fired at different temperatures.

In general, a high drop in the apparent porosity is observed after firing above 1150 °C for all samples is due to the presence of impurities such as iron and alkali (Na_2_O and K_2_O) oxides in the bypass and silica fume. This amount of alkali leads to the formation of feldspar, which tends to form in the liquid phase above 1100 °C. This liquid phase fills the open pores between the grain boundaries, enhances the densification parameters, and changes the sample's color from light to dark brown at high temperatures^5,74,75^, as seen in Figs. 20 and 21.

Wollastonite was made by Rashita Abd Rashid et al.^80^, utilizing a solid-state reaction with limestone and silica sand. The samples were burned at 1450 °C with limestone to silica sand ratios of 1:1, 2:1, and 3:1. It was found that wollastonite is visible at an early ratio for a 1:1 fire at 1450 °C. However, additional phases, such as olivine and larnite, were noted in 2:1 and 3:1 ratios. According to Chen et al.^87^ and Wesselsky and Jensen^88^, the phases exist at greater molar ratios of silica sand to limestone, specifically 2:1 and 3:1. The wollastonite phase was absent at the 2:1 and 3:1 molar ratio. The sintering temperature and raw material purity had a significant impact on the density of the colored products at a molar ratio of 1:1. Wollastonite was generated at a temperature close to its usual density of 2.86–3.09 g cm^−3^, following sintering at 1450 °C.

### Compressive strength

**Fig. 22:**
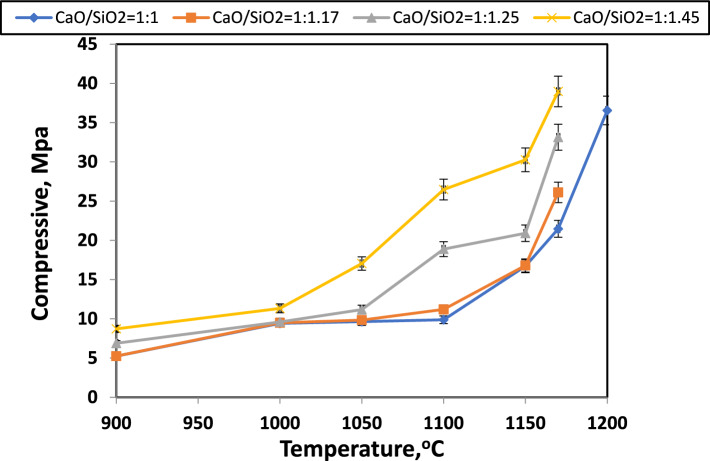
Compressive strength of samples with different CaO/SiO_2_ ratios fired at different temperatures.

Figure 22 illustrates the compressive strength of samples at different firing temperatures for different CaO/SiO_2_ ratios. An increase in silica content in the samples was observed to increase compressive strength to reach 40 MPa for the samples with a CaO/SiO_2_ ratio of 1:1.45. Among the samples, the samples with CaO/SiO_2_ = 1:1 had the lowest strength at 1170 °C compared to other samples. Furthermore, it was observed that the increase in firing temperature decreased porosity because of improved particle interaction, leading to an enhancement of the compressive strength with firing temperature^81,83^. Numerous researchers concurred that porosity, influenced by particle size and distribution, is directly related to compressive strength^81,89^. A similar discovery suggested that a finer particle size could increase compressive strength (or decrease porosity)^81,90^. The grains grow between the fine particles and eventually bond them together under the impact of particle dissolving action and high temperature. Pores are sealed off, and porosity gradually diminishes as grain boundaries expand. Furthermore, the increased formation of vitreous phases, which close the pores and improve the samples' strength and density, reduced porosity and a higher degree of calcic phases (wollastonite)^91^.

The maximum strength obtained in this work is like of Srinath Palakurthy et al.^92^, who prepared β-wollastonite by using natural waste materials such as rice husk ash (RHA) and eggshells based on using sol–gel method and sintered the sample at 850 °C. It is demonstrated that the mechanical properties revealed excellent compressive strength (40.77 ± 2.46 MPa).

Table 6 compares the results for apparent porosity, bulk density, and compressive strength from the current study with those from previous studies in the literature.

**Table 6:** Comparison of the apparent porosity, bulk density, and compressive strength results from this study with those reported in previous literature.

Precursors for preparation of wollastonite	Synthesis method	Synthesis temperature (°C)	Physical Properties		
			Bulk density (g/cm^3^)	Porosity %	Compressive strength (MPa)
Bypass and silica fume (present work)	Temperature induced forming technique	1170	2.2	8	40
Eggshell waste and commercial SiO_2_^93^	Microwave assisted solid-state reaction	1100	2.01	29	–
Rice husk ash and cement kiln dust^94^	Solid-state reaction	1100	1.4	43	50
Eggshell and waste glass^1^	Solid-state reaction	900	2.90	–	–
Waste soda-lime-silica glasses^95^	control crystallization sintering process	1000	2–2.2	0.65–0.7	–
Silica sand and limestone^80^	Solid-state reaction	1450	2.86–3.09	–	–
Eggshells and rice husk ash^76^	Solid-state reaction	1200	2.562	4.23	–
Rice husk and eggshells^92^	Sol–gel method	850	–	–	40.77 ± 2.46

## Conclusion

- Pure systems, utilizing a composition with a CaO/SiO_2_ ratio of 1:1, failed to produce wollastonite at all temperatures ranging from 900 to 1200 °C.
- The presence of impurities in alkalis, Fe_2_O_3_, and MgO in both bypass and silica fume wastes resulted in larnite and rankinite, with a small amount of wollastonite.
- To obtain wollastonite as the single phase, the silica content needs to be increased beyond the stoichiometric composition. The most favorable molar ratio of CaO/SiO_2_ for the current wastes is determined to be 1:1.45.
- The composition with CaO/SiO_2_ of 1:1.17 and 1:1.25 still had larnite beside the wollastonite formation up to 1100 °C.
- Increasing the firing temperature above 1100 °C to 1170 °C for composition of CaO/SiO_2_ molar ratios of 1:1.17 and 1:1.25 showed the formation of pseudo-wollastonite with larnite.
- When the CaO/SiO_2_ ratio is increased to 1:1.45, primarily pure wollastonite is observed, with minimal larnite, especially as the firing temperature is raised to 1100 °C.
- Wollastonite changed into pseudo-wollastonite after firing above 1100 °C for a CaO/SiO_2_ ratio of 1:1.45.
- Increasing the silica content in the stoichiometric composition for wollastonite formation, the decrease in the apparent porosity and the increase in the bulk density was observed to reach 8% and 2.2 g/cm^3^ for CaO: SiO_2_ of 1:1.45 at 1170 °C respectively. Increasing the silica content in the stoichiometric composition for wollastonite formation resulted in a decrease in apparent porosity and an increase in bulk density, reaching 8% and 2.2 g/cm^3^, respectively, for a CaO: SiO_2_ ratio of 1:1.45 at 1170 °C.
- The maximum compressive strength of about 40 MPa was obtained for samples with high silica contents at 1170 °C.
- The present study produced mainly pure wollastonite ceramic bodies by increasing the silica content to overcome impurities in the utilized wastes. As a result, this expansion enhances the potential applications of ceramic products, including their use in heat-insulating ceramics, packaging, foundry lining, and as a filler in paints and polymers within the metallurgy and automotive industries.

## Data Availability

The data sets generated and/or analyzed during the current study are not publicly available because they are for authors but are available from the corresponding author on reasonable request.
